# Three-Dimensional Dynamic Cell Models for Metabolic Dysfunction-Associated Steatotic Liver Disease Progression

**DOI:** 10.34133/bmef.0181

**Published:** 2025-09-30

**Authors:** Zhengxiang Huang, Lili Li, Kevin Dudley, Lan Xiao, Gary Huang, V. Nathan Subramaniam, Chen Chen, Ross Crawford, Yin Xiao

**Affiliations:** ^1^ School of Medicine and Dentistry, Griffith University, Gold Coast, QLD 4222, Australia.; ^2^ Institute for Biomedicine and Glycomics, Griffith University, Southport, QLD 4222, Australia.; ^3^ School of Biomedical Sciences, University of Queensland, St Lucia, QLD 4072, Australia.; ^4^ Nanjing Stomatological Hospital, Affiliated Hospital of Medical School, Research Institute of Stomatology, Nanjing University, Nanjing, China.; ^5^ Central Analytical Research Facility, Queensland University of Technology, Brisbane, QLD 4000, Australia.; ^6^ Hepatogenomics Research Group, Centre for Genomics and Personalised Health, School of Biomedical Sciences, Queensland University of Technology, Brisbane, QLD 4059, Australia.; ^7^ Orthopaedic Research Unit, School of Mechanical, Medical & Process Engineering, Faculty of Engineering, Queensland University of Technology, Brisbane, QLD 4000, Australia.; ^8^ Centre for Biomedical Technologies, Queensland University of Technology, Brisbane, QLD 4000, Australia.

## Abstract

**Objective:** Metabolic dysfunction-associated steatotic liver disease (MASLD) is a complex, progressive disorder involving multiple cell types, ranging from simple steatosis to metabolic dysfunction-associated steatohepatitis (MASH), characterized by pro-inflammatory macrophage activation, and can eventually advance to fibrosis, initiated by hepatic stellate cells (HSCs). In vitro multi-cell coculture models are vital tools for elucidating the mechanisms underlying MASLD. **Impact Statement:** Existing in vitro models for MASLD, including traditional 2-dimensional (2D) cultures and advanced organ-on-a-chip and organoid systems, face challenges in representing multiple cell types and analyzing them individually. Here, utilizing a cell carrier developed in our laboratory, we introduce a series of 3D dynamic coculture models that simulate different stages of MASLD progression and enable individual cell type analysis. **Introduction:** Currently, no single system provides an optimal balance of control, reproducibility, and analytical convenience. Most in vitro models lack the ability to isolate and analyze individual cell types post-culture, making it difficult to study cell-specific responses in MASLD progression. **Methods:** The 3D hollow porous sphere cell carrier allows cells to grow on its surface, while the culture device (mini-bioreactor) creates a dynamic environment. The 3 distinct MASLD models were established based on cocultured cell types: steatosis (hepatocytes only), MASH (hepatocytes and macrophages in a 4:1 ratio), and fibrosis (hepatocytes, macrophages, and HSCs in an 8:2:1 ratio). Well-established MASLD mouse models were employed to validate our in vitro 3D dynamic MASLD models, using 7-week-old male C57BL/6J mice fed a high-fat diet. **Results:** Our models demonstrate a progressive decline in hepatocyte viability and increased lipid accumulation, mirroring in vivo pathology. Additionally, gene expression profiles of our models align with those observed in MASLD-affected mouse livers. Notably, comparative analysis highlights the role of pro-inflammatory macrophages in disrupting hepatocyte lipid metabolism. **Conclusion:** These models offer a robust platform for investigating MASLD mechanisms and show potential for screening anti-MASLD therapeutics.

## Introduction

Metabolic dysfunction-associated steatotic liver disease (MASLD) represents a prevalent chronic condition, affecting approximately 30% of the global population [1]. MASLD is characterized by the accumulation of lipids in the liver, exceeding 5%, in the absence of substantial alcohol consumption. The disease progresses through several stages [2]: (a) steatosis, marked by the initial accumulation of lipids in hepatocytes, typically without pronounced symptoms; (b) metabolic dysfunction-associated steatohepatitis (MASH), involving the recruitment and activation of macrophages into a pro-inflammatory state, leading to the secretion of pro-inflammatory cytokines and subsequent liver cell damage; (c) fibrosis, where hepatic stellate cells (HSCs) become activated, producing collagen and resulting in fibrosis and liver cirrhosis; and (d) hepatocellular carcinoma (HCC), the terminal stage characterized by a remarkably reduced survival rate of the patient.

In vitro models serve as essential tools for elucidating the mechanisms underlying MASLD. Given the critical role of elevated free fatty acids (FFAs) in MASLD pathogenesis [3], most in vitro models simulate the disease by incorporating high concentrations of FFAs in the culture media to induce lipid accumulation in hepatocytes [4]. The conventional and widely accepted in vitro model for MASLD is the 2-dimensional (2D) hepatocyte monoculture system [5], such as culturing hepatocytes in a tissue culture plates. Although this 2D monoculture effectively replicates certain MASLD aspects, such as lipid accumulation during simple steatosis, it lacks interactions with other cell types, failing to represent more advanced stages like MASH and fibrosis.

In recent years, innovative in vitro MASLD models have emerged, leveraging technologies like microfluidics/organ-on-a-chip [6–8] and spheroids/organoids/microtissues [9–12]. These models incorporate multiple cell types (hepatocytes, macrophages, HSCs, and endothelial cells) and offer varying degrees of physiological relevance and scalability, while studies have yet to compare these in vitro models with in vivo models or traditional 2D systems. Currently, no single system provides an optimal balance of control, reproducibility, and analytical convenience. Spheroids and organoids have limited control over cell composition and metabolic gradients. Microfluidic and organ-on-a-chip systems are highly sophisticated, but their cost and operational complexity hinder their widespread use in routine research and drug screening. The complexity of these models often makes them challenging to establish, and the development of MASLD phenotypes typically requires more than 10 d, posing practical difficulties. Moreover, most in vitro models lack the ability to isolate and analyze individual cell types post-culture, making it difficult to study cell-specific responses in MASLD progression.

This study introduces a 3D dynamic system that replicates various MASLD stages based on a previously developed cell carrier [13]. This includes (a) steatosis (hepatocyte monoculture), (b) MASH (hepatocytes and macrophages), and (c) fibrosis (hepatocytes, macrophages, and HSCs). We validated the expression of vital metabolic-related genes in this model, comparing them with histologically verified MASLD mouse models. In addition, our model enables the discovery of the cell–cell interactions occurring during MASLD progression. Remarkably, our model requires only 1 d to develop a MASLD phenotype, allowing for the isolation and analysis of individual cell types. This innovative approach promises to be a valuable tool for studying cell–cell interactions in MASLD and for exploring the mechanisms of action of potential anti-MASLD drugs.

## Materials and Methods

### Cell culture

In this study, Huh7 [PTA-4583, American Type Culture Collection (ATCC), Manassas, VA, USA], THP1 (TIB-202, ATCC, Manassas, VA, USA), and LX2 (SCC064, Millipore Sigma, Burlington, MA, USA) cell lines were utilized to represent hepatocytes, macrophages, and HSCs, respectively. Huh7 cells originate from a well-differentiated HCC, THP1 cells are derived from a human acute monocytic leukemia patient, and LX2 cells are an immortalized human HSC line. Huh7 cells were maintained in Dulbecco’s modified Eagle’s medium (DMEM) (11885, Thermo Fisher Scientific) supplemented with 10% fetal bovine serum (FBS). THP1 cells were grown in RPMI 1640 (A10491, Thermo Fisher Scientific) with 10% FBS, while LX2 cells were cultured in DMEM high glucose (11995, Thermo Fisher Scientific) containing 2% FBS. All cell cultures were incubated at 37 °C with 5% CO_2_.

### Experimental design

**Fig. 1.**
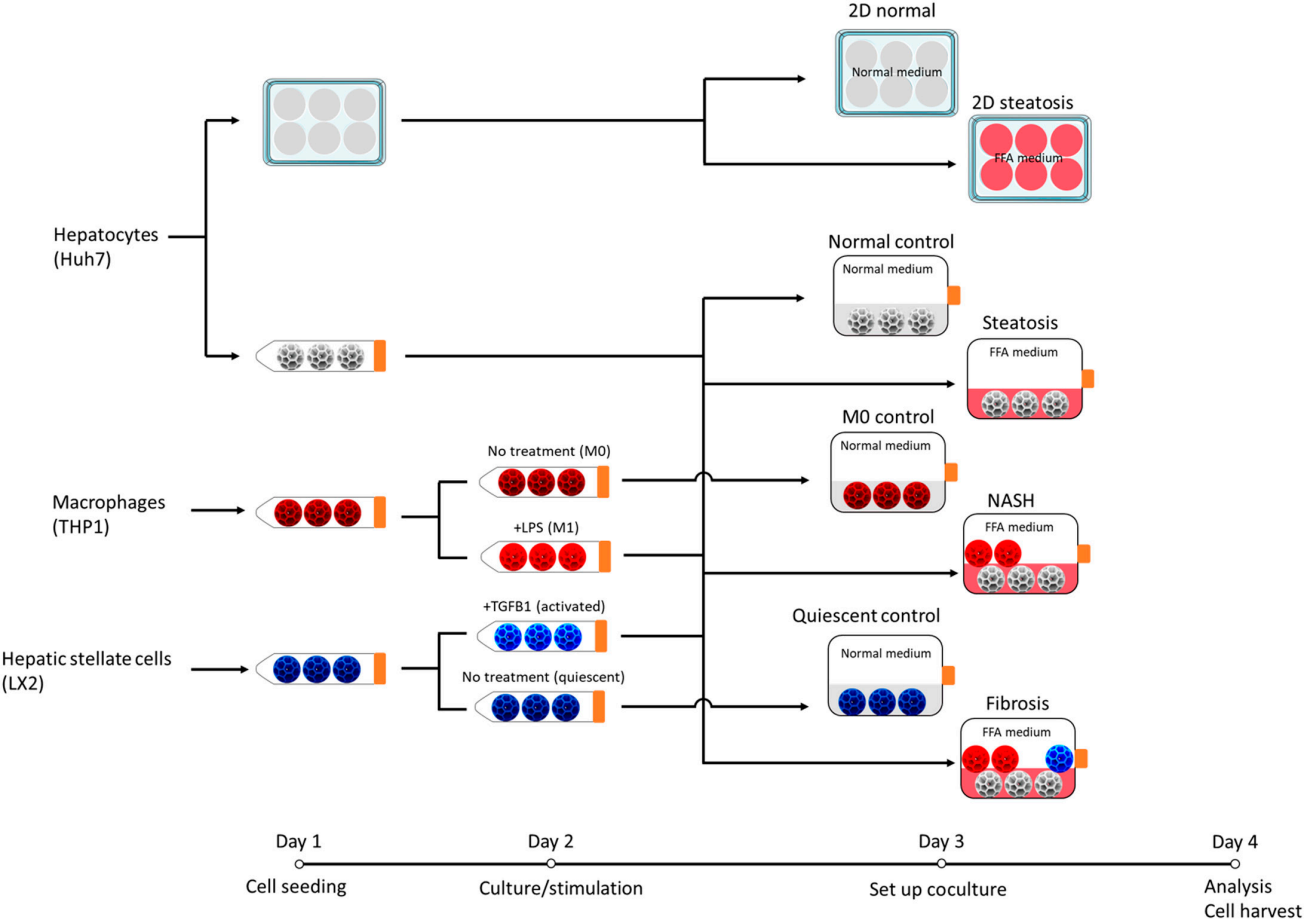
Flowchart illustrating the experimental design.

The overarching experimental design is depicted in Fig. 1. On day 1, each cell type was seeded on uniquely colored cell carriers (Huh7 on white, THP1 on red, and LX2 on blue). On day 2, the Huh7 cells underwent continued culturing, whereas THP1 cells were stimulated with lipopolysaccharide (LPS) and LX2 cells were treated with transforming growth factor-β1 (TGF-β1) for 24 h. On day 3, the 3 different MASLD models were established based on the cocultured cell types: steatosis (hepatocytes only), MASH (hepatocytes and macrophages in a 4:1 ratio), and fibrosis (hepatocytes, macrophages, and HSCs in an 8:2:1 ratio) with reference to previous studies [9,14,15]. After 24 h of coculturing, the different cell types were harvested individually for further analysis.

More specifically, cell carriers were fabricated and modified in the laboratory using a previously established method [13]. Briefly, the 3D hollow porous sphere cell carrier allows the cells to grow on its surface, and the culture device (mini-bioreactor) creates a dynamic environment. To initiate seeding on day 1, cell suspensions and carriers were combined in 15-ml centrifuge tubes. For THP1 cells, 100 nM phorbol 12-myristate 13-acetate (PMA) (Sigma, P8139) was added to induce differentiation into adherent macrophages. The tubes were then placed on a Tube Roller (Thermo Scientific) at 1.5 rpm for 24 h. On day 2, cell carriers were washed with phosphate-buffered saline (PBS), transferred to 50-ml mini-bioreactors (431720, Corning) containing respective culture media, and cultured at 5 rpm for another 24 h. During this time, half of the THP1 macrophages were stimulated with 100 ng/ml LPS (L2630, Sigma-Aldrich) to induce a pro-inflammatory M1 phenotype, while the remainder were cultured in LPS-free medium to maintain an unstimulated M0 phenotype. Half of the LX2 cells were similarly stimulated with 25 ng/ml TGF-β1 (Promega) to induce an activated state, with the rest cultured in the standard medium as a quiescent state. On day 3, all cell carriers were washed with PBS and arranged in mini-bioreactors for coculturing as per the group allocations. The culture medium for this stage was DMEM (11885, Thermo Fisher Scientific) with 2% FBS. In the MASLD groups (steatosis, MASH, and Fibrosis), 0.25 mM FFAs (a mixture of oleic acid and palmitic acid at a 2:1 ratio) were added. After 24 h of culturing, on-site analyses (live/dead and lipid staining) were performed, and cells were also harvested for RNA sequencing (RNA-seq) and quantitative polymerase chain reaction (qPCR). To compare our 3D dynamic MASLD models with traditional 2D models, hepatocytes were cultured in classic 6-well plates using the same media as in the cell carrier models. All experiments were performed with 3 biological replicates, each consisting of 2 technical replicates.

### Animal MASLD models

We employed well-established MASLD mouse models to validate our in vitro 3D dynamic MASLD models utilizing 7-week-old male C57BL/6J mice with a high-fat diet feeding [16]. These mice were accommodated at a controlled temperature (22 ± 2 °C) and subjected to a 12-h light/dark cycle within the Institute for Bioengineering and Nanotechnology at the University of Queensland. Mice had unrestricted access to both water and food. MASLD phenotypes were induced by administering a high-fat diet (43% caloric content from fat, SF04-001, Specialty Feeds, Glen Forrest, WA, Australia) starting from the age of 8 weeks. Mice maintained on a standard diet served as healthy controls. For the development of steatosis and MASH models (*n* = 5 for steatosis model, *n* = 7 for MASH model), mice were euthanized after 16 and 28 weeks (corresponding to 24 and 35 weeks of age), respectively. In the fibrosis model (*n* = 5), mice were administered 50 mg/kg streptozotocin (STZ; Wako) intraperitoneal injections over 3 consecutive days at 14 weeks of age, followed by continued high-fat diet consumption until euthanasia at 35 weeks. The STZ treatment, in conjunction with the high-fat diet, expedited the progression of MASLD to the fibrosis stage [17]. Collected mouse livers were either preserved in 4% paraformaldehyde (PFA) for histological analysis or snap-frozen for molecular studies. All experimental protocols were approved by the University of Queensland Animal Ethics Committee (2020/AE000231).

To ascertain the stages of MASLD in the mouse livers, optimal cutting temperature (OCT)-embedded livers were sectioned at 10-μm thickness and subjected to hematoxylin and eosin (H&E), Oil Red O, and Picrosirius Red staining. Imaging was performed using a Nikon light microscope.

### Cell viability assay

Cell viability was assessed for each cell carrier using the Alamar Blue Cell Viability Reagent (DAL1025, Invitrogen), as per the manufacturer’s instructions. Each cell carrier was placed in a 24-well plate and incubated with 2 ml of cell culture medium containing 10% Alamar Blue reagent for 2 h. The absorbance was then measured using a fluorescence-based plate reader (BMG Omega, BMG LABTECH; excitation: 540 nm, emission: 590 nm). To control for variations in cell numbers across carriers within the same group, the Alamar Blue assay was conducted twice: immediately post-seeding (day 2) and at the end of the experiments (day 4). Results were expressed as the fold change ratio between day 4 and day 2.

### Live/dead staining

Live and dead cells on the cell carrier were visualized using fluorescein diacetate (FDA; F1303, Thermo Fisher Scientific) and propidium iodide (PI; P1304MP, Thermo Fisher Scientific) staining. The cell carrier was incubated in a base cell culture medium (without FBS) with 5 mg/ml FDA and 2 mg/ml PI for 5 min at room temperature, followed by 2 PBS washes. Imaging was conducted using a Nikon SMZ25 stereo microscope.

### Intracellular lipid staining and quantification

Intracellular lipid content in Huh7 cells was visualized and quantified through Oil Red O staining of the cell carrier. The Oil Red O working solution was prepared by combining 3 parts of the Oil Red O stock solution (3 mg/ml in isopropanol) with 2 parts deionized water. Cells were cleansed twice with PBS, fixed in 4% PFA for 15 min, rinsed once with 60% isopropanol, and stained with the Oil Red O working solution for 15 min, followed by 3 washes with deionized water. Imaging was again performed using a Nikon SMZ25 stereo microscope. The Oil Red O stain was extracted from each cell carrier using 500 μl of isopropanol for quantitative analysis. Absorbance was measured at 492 nm using the ClarioStar microplate reader and normalized to the total DNA content, as determined by the PicoGreen DNA assay. This normalized value represents the average lipid content per cell.

### RNA extraction, cDNA synthesis, and real-time qPCR

Total RNA from cells in the cell carrier and mouse livers was extracted using TRIzol, followed by purification with a PureLink RNA Mini Kit (Thermo Fisher Scientific). cDNA synthesis was conducted using the SensiFAST cDNA Synthesis Kit (Bioline). qPCR was carried out using SYBR Green PCR Master Mix (Thermo Fisher Scientific) on a QuantStudio 7 Real-Time PCR System (Thermo Fisher Scientific). Primer pairs are detailed in the Supplementary Materials (Tables S1 and S2). The expression level of the target gene was quantified as a fold change relative to the housekeeping gene (β-actin) using the 2−ΔΔCT method.

### RNA-seq

RNA concentration and integrity were assessed using the Nanodrop and Agilent Fragment Analyzer 5200, following the manufacturer’s guidelines. Samples with an RNA integrity number of at least 7.0 were selected for transcriptomic analysis. For each sample, 250 ng of RNA was submitted to Expression Analysis. Illumina-based deep sequencing yields approximately 20 million 50-base pair paired-end reads. Post-sequencing, reads were pseudoaligned to either the human (GRCh38) or mouse (GRCm39) transcriptome, and transcript quantification was performed using kallisto. Gene-level estimates were derived using the tximport package in R. Differential expression analysis compared raw read count data between the disease model and its control using the R package DESeq2. Differentially expressed genes (DEGs) were identified with a false discovery rate (FDR) of <5%. Aiming for hepatocyte-specific gene expression data from mouse livers, a filter (baseMean > 2,000) was applied to the mouse livers, since nearly 80% of the cells in the liver are hepatocytes [18]. Identified DEGs were analyzed using WebGestalt (WEB-based Gene SeT AnaLysis Toolkit) (https://www.webgestalt.org/) for pathway enrichment, with significant pathways defined by an FDR of <5%.

### Statistical analysis

Data analysis was conducted using GraphPad Prism 9 software. Results were presented as scatterplots, showing the mean ± standard error of the mean (SEM). Group differences were evaluated using 2-tailed Student’s *t* test and one-way analysis of variance (ANOVA), with a *P* value of <0.05 considered statistically significant.

## Results

In assessing the biocompatibility of our newly developed system with Huh7 (human HCC cell line 7), THP1 (human monocytic leukemia cell line 1), and LX2 cells (human HSC line 2), FDA/PI staining was performed on cell carriers seeded with these cells. As shown in Fig. S1, the findings revealed effective seeding and uniform distribution of all 3 cell types on the cell carriers. Predominantly, cells were stained green, signifying their viability under standard culture conditions. In the MASH model and fibrosis model, macrophages and HSCs also exhibited stable viability, supporting the suitability of these cells (Fig. S2).

**Fig. 2.**
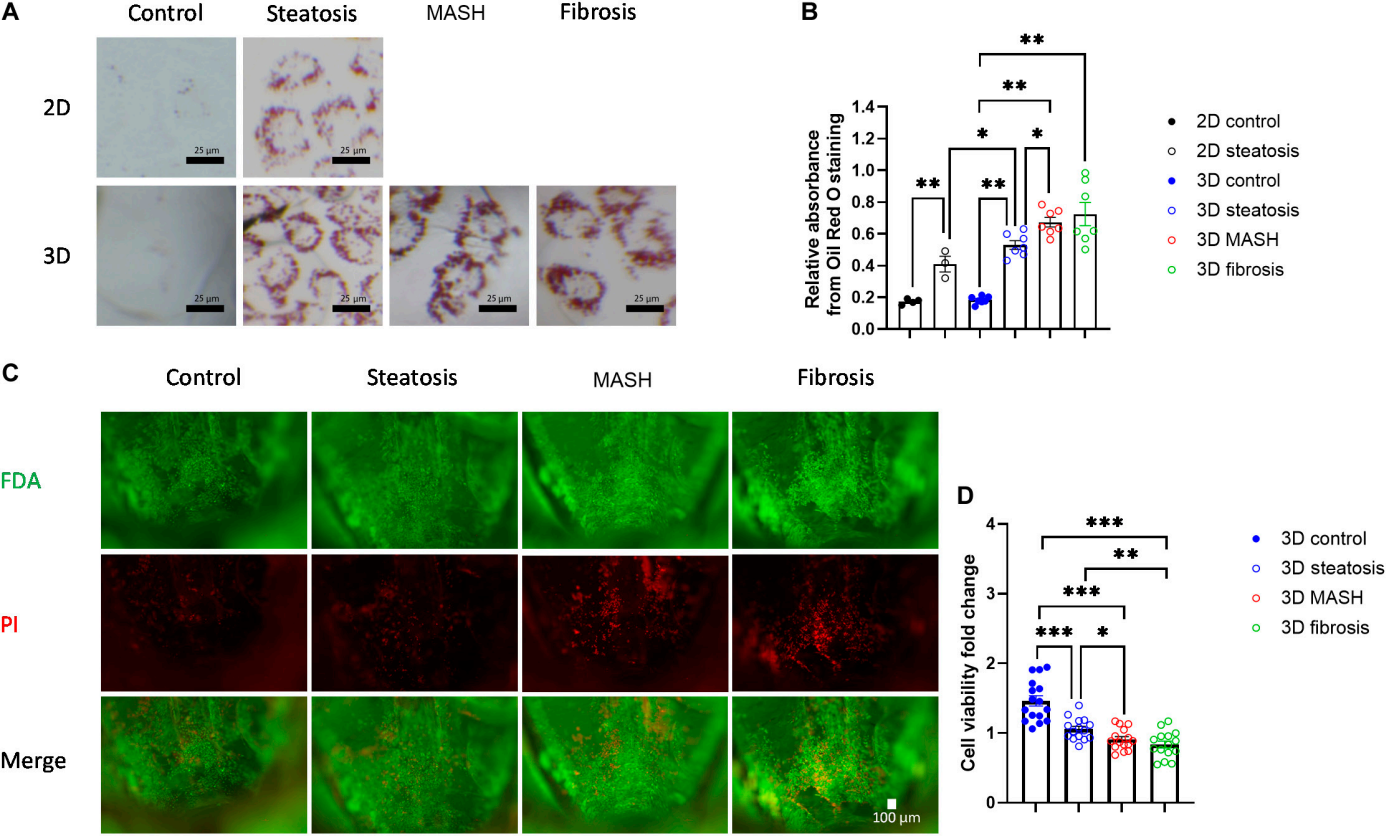
Phenotypic changes of hepatocytes in our 3D dynamic MASLD models. (A) Oil Red O staining of hepatocytes cultured on the 2D 6-well plates or 3D cell carriers. (B) Relative Oil Red O absorbance corrected by total DNA content of hepatocytes on a 2D 6-well plate or 3D cell carriers. (C) FDA/PI staining for hepatocytes on the 3D cell carriers. (D) Fold change of the fluorescent intensity from the Alamar Blue cell viability assay. Data are presented as a scatterplot with mean ± SEM. *P < 0.05, **P < 0.01.

The progression of MASLD typically involves incremental lipid accumulation and cellular damage in hepatocytes. To evaluate these aspects, we employed Oil Red O staining with quantification, FDA/PI staining, and the Alamar Blue cell viability assay on hepatocytes in the cell carriers of the steatosis, MASH, and fibrosis models. The findings indicated augmented intracellular lipid accumulation in both traditional 2D (6-well plate) and novel 3D cell carrier-based MASLD models (Fig. 2A and B), with the 3D models demonstrating a more pronounced increase. Compared with the 2D model, intracellular lipid accumulation increased by 14.7% in the steatosis model, 45.8% in the MASH model, and 56.7% in the fibrosis model. Notably, in the 3D models of steatosis, MASH, and fibrosis, there was a progressive increase in intracellular lipid accumulation (Fig. 2A and B), effectively simulating the pathologic lipid accumulation observed during MASLD progression. Concurrently, there was a gradual decline in hepatocyte viability in the 3D models of steatosis, MASH, and fibrosis (Fig. 2C and D), mirroring the progressive cell damage and death observed in MASLD progression in vivo [19].

We established mouse models representing different stages of MASLD (steatosis, MASH, and fibrosis) to evaluate whether and to what extent our 3D in vitro MASLD model can replicate the in vivo conditions. Representative images are presented in Figs. S3 to S5. H&E staining revealed progressive hepatic pathology from steatosis to MASH and fibrosis. Steatosis was characterized by macrovesicular and microvesicular lipid droplet accumulation, with clear cytoplasmic vacuoles and mild hepatocyte enlargement confirmed by abundant red-stained lipid droplets in Oil Red O staining (Fig. S3). In MASH, H&E staining demonstrated hepatocyte ballooning, inflammatory infiltration, and cellular injury, with lipid accumulation in Oil Red O staining (Fig. S4). Fibrosis exhibited pronounced hepatocyte degeneration and disrupted hepatic architecture in H&E staining, with lipid accumulation in Oil Red O staining, and extensive collagen deposition with fibrotic septa bridging portal and central regions in Picrosirius Red staining, indicating advanced fibrosis (Fig. S5).

Considering that the predominant changes in hepatocytes during MASLD involve aberrant lipid metabolism, the expression of a set of critical genes involved in lipid metabolism, lipid biosynthesis [*SREBP1* (*sterol regulatory element-binding protein 1*) and *FASN* (*fatty acid synthase*)] and uptake [*CD36* (*cluster of differentiation 36*)], oxidation [*PGC1A* (*peroxisome proliferator-activated receptor gamma coactivator 1-alpha*) and *CPT1A* (*carnitine palmitoyltransferase 1a*)], and glucose uptake [*GLUT2* (*glucose transporter type 2*)] was analyzed. We measured their expression levels in hepatocytes across 2D, 3D, and mouse MASLD models. Additionally, given the importance of inflammation and fibrous tissue formation in the MASH and fibrosis stages, which were initiated by macrophages and HSCs, we also quantified these changes in macrophages and HSCs in the 3D MASH and fibrosis models, as well as in corresponding animal models.

**Fig. 3.**
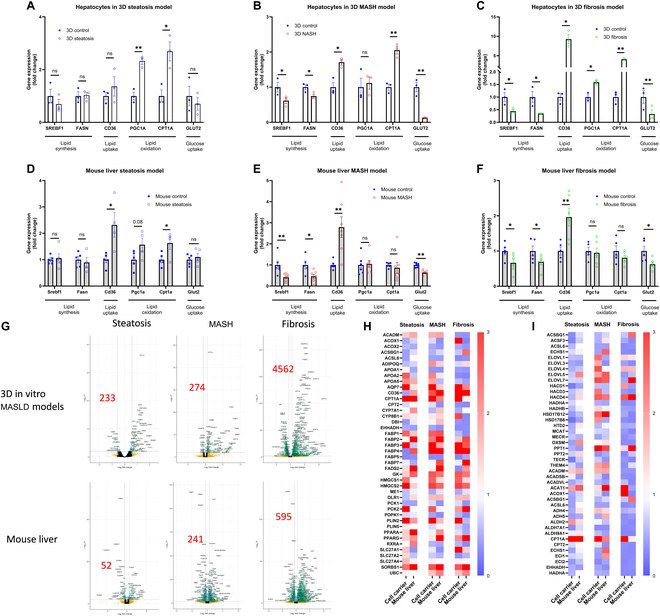
Transcriptional characterization of hepatocytes in our 3D dynamic models and mouse livers for MASLD. (A to F) Gene expression fold change was measured by qPCR for hepatocytes in our 3D (A) steatosis, (B) MASH, and (C) fibrosis models, and for mouse livers with (D) steatosis, (E) MASH, and (F) fibrosis. Volcano plots (G) showing DEGs in the MASLD groups compared to their relative control groups. Heatmaps (H and I) show KEGG-annotated genes from PPAR signaling (ko03320) (H) and from fatty acid biosynthesis (ko00061), elongation (ko00062), and degradation (ko00071) (I). Genes shown are those significant in at least one comparison (DESeq2 FDR < 0.05). Data are presented as a scatterplot with mean ± SEM. *P < 0.05, **P < 0.01. *N* = 3 for in vitro models. *N* = 5 (steatosis), *N* = 7 (MASH), and *N* = 5 (fibrosis) for in vivo models (mice).

In the steatosis models, hepatocytes of the 3D group exhibited no notable alterations in the expression of genes associated with lipid synthesis and glucose uptake. Conversely, there was an up-regulation in the expression of genes linked to lipid oxidation. These changes in gene expression paralleled those observed in the mouse steatosis model (Fig. 3A and D). Similarly, within the MASH models, the 3D group’s hepatocytes demonstrated a decrease in lipid synthesis and glucose uptake gene expression, coupled with an elevation in lipid uptake gene expression. These changes were akin to those in the MASH mouse model (Fig. 3B and E). In the 3D fibrosis models, hepatocytes mirrored this pattern, showing diminished lipid synthesis and glucose uptake gene expression and augmented lipid uptake gene expression, thus replicating the gene expression profiles observed in the fibrosis mouse model (refer to Fig. 3C and F). Collectively, these results suggest that the 3D steatosis, MASH, and fibrosis models reflect most of the principal gene expression patterns in lipid metabolism found in their respective animal models.

Furthermore, we assessed changes in lipid metabolism gene expression in the steatosis model using a conventional 2D cell culture system (Fig. S6). We compared these findings with those from the 3D and mouse steatosis models. The analysis revealed that the 3D steatosis model more closely emulated the lipid metabolic gene expression patterns seen in the animal steatosis model compared to the traditional 2D model. Notably, SREBP1 and FASN did not change significantly in the 3D and mouse steatosis models, whereas PGC1A/CPT1A increased in both, consistent with an early metabolic response favoring fatty acid oxidation over robust repression of de novo lipogenesis (Table 1).

**Table 1.** The gene expression changes in the 2D, 3D, and mouse steatosis models compared to their relative controls

	2D steatosis		3D steatosis		Mouse steatosis	
Gene	Direction	*P*	Direction	*P*	Direction	*P*
*SREBP1*	↓	<0.01	↔	0.38	↔	0.78
*FASN*	↓	<0.05	↔	0.89	↔	0.64
*CD36*	↓	0.06	↔	0.40	↑	<0.05
*PGC1A*	↑	<0.01	↑	<0.01	↑	0.08
*CPT1A*	↑	<0.05	↑	<0.05	↑	<0.05
*GLUT2*	↔	0.80	↔	0.53	↔	0.54

To gain deeper insight into the gene expression profiles of hepatocytes, we conducted RNA-seq in both the 3D dynamic and animal models of MASLD. The resulting volcano plot (Fig. 3G) illustrated that in the mouse MASLD models, there was a progressive increase in the number of DEGs correlating with the advancement of MASLD (from steatosis to MASH to fibrosis). Notably, hepatocytes in the 3D MASLD models successfully mirrored this trend (Fig. 3G).

Given the pivotal role of the peroxisome proliferator-activated receptor (PPAR) pathway in regulating lipid metabolism, we selected a list of genes from the PPAR pathway and those involved in fatty acid metabolism (encompassing biosynthesis, elongation, and degradation) based on the Kyoto Encyclopedia of Genes and Genomes (KEGG) database. Our findings revealed a striking similarity in the gene expression profiles within the PPAR pathway (Fig. 3H) and fatty acid metabolic processes (Fig. 3I) between the 3D models and the animal models across the steatosis, MASH, and fibrosis stages. These results demonstrate that our 3D dynamic MASLD models are able to represent the major gene expression profile of lipid metabolism observed in the MASLD mouse livers.

**Fig. 4.**
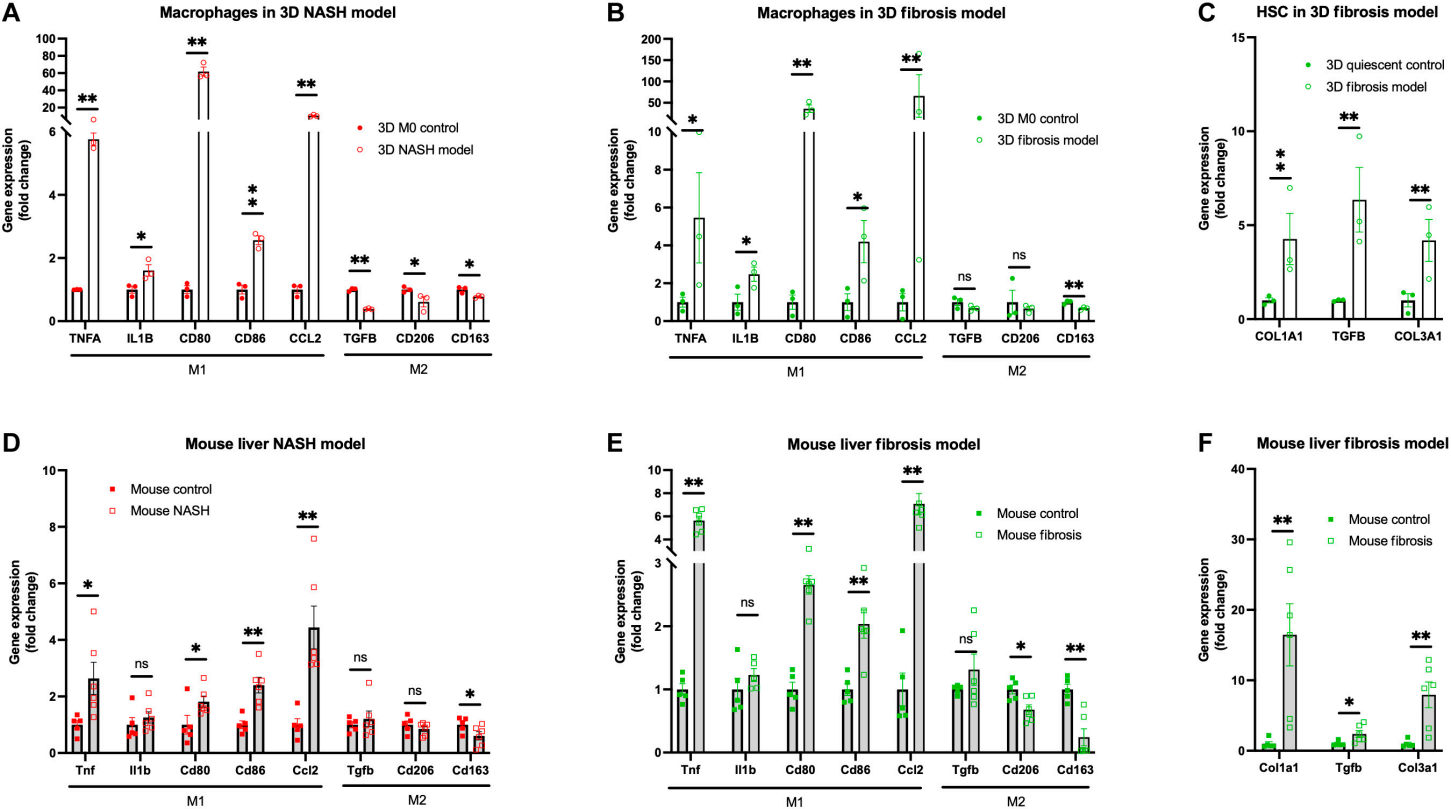
Gene expression of nonparenchymal cells in 3D in vitro and mouse MASLD models. Gene expression fold change was measured by qPCR for macrophages from the (A) 3D MASH model, (B) 3D fibrosis model, (C) HSCs from the 3D fibrosis model, (D) mouse liver of MASH model, and (E and F) mouse liver of fibrosis model (E and F). Data are presented by scatterplot with mean ± SEM. *P < 0.05, **P < 0.01. *N* = 3 for in vitro models. *N* = 7 (MASH) and *N* = 5 (fibrosis) for in vivo models (mice).

Nonparenchymal cells (NPCs), including macrophages and HSCs, contribute significantly to the MASLD disease phenotypes, i.e., inflammation and fibrous tissue formation [20,21]. To validate these phenotypes, qPCR was employed to examine the expression of genes associated with inflammation and fibrotic tissue development. In the 3D MASH model, macrophages were isolated, and the expression levels of pro-inflammatory M1 and anti-inflammatory M2 markers were quantified through qPCR. These levels were then compared with those in mouse MASH livers. Our findings indicated a significant up-regulation of M1 markers and a down-regulation of M2 markers in macrophages within the 3D MASH model, closely resembling the alterations observed in MASH-affected mouse livers (Fig. 4A and D). Similarly, in the 3D fibrosis model, macrophages and HSCs were isolated. The macrophages exhibited a predominant M1 pro-inflammatory state (Fig. 4B), mirroring the phenotype observed in fibrotic mouse livers (Fig. 4E). The HSCs demonstrated a marked up-regulation in gene expression associated with fibrous tissue formation, as compared to their quiescent counterparts (Fig. 4C). This up-regulation paralleled the gene expression changes seen in fibrotic mouse livers (Fig. 4F). These results of macrophages and HSCs affirm that the 3D MASH and fibrosis models effectively replicate key aspects of NPC behavior during MASLD progression in vivo.

**Fig. 5.**
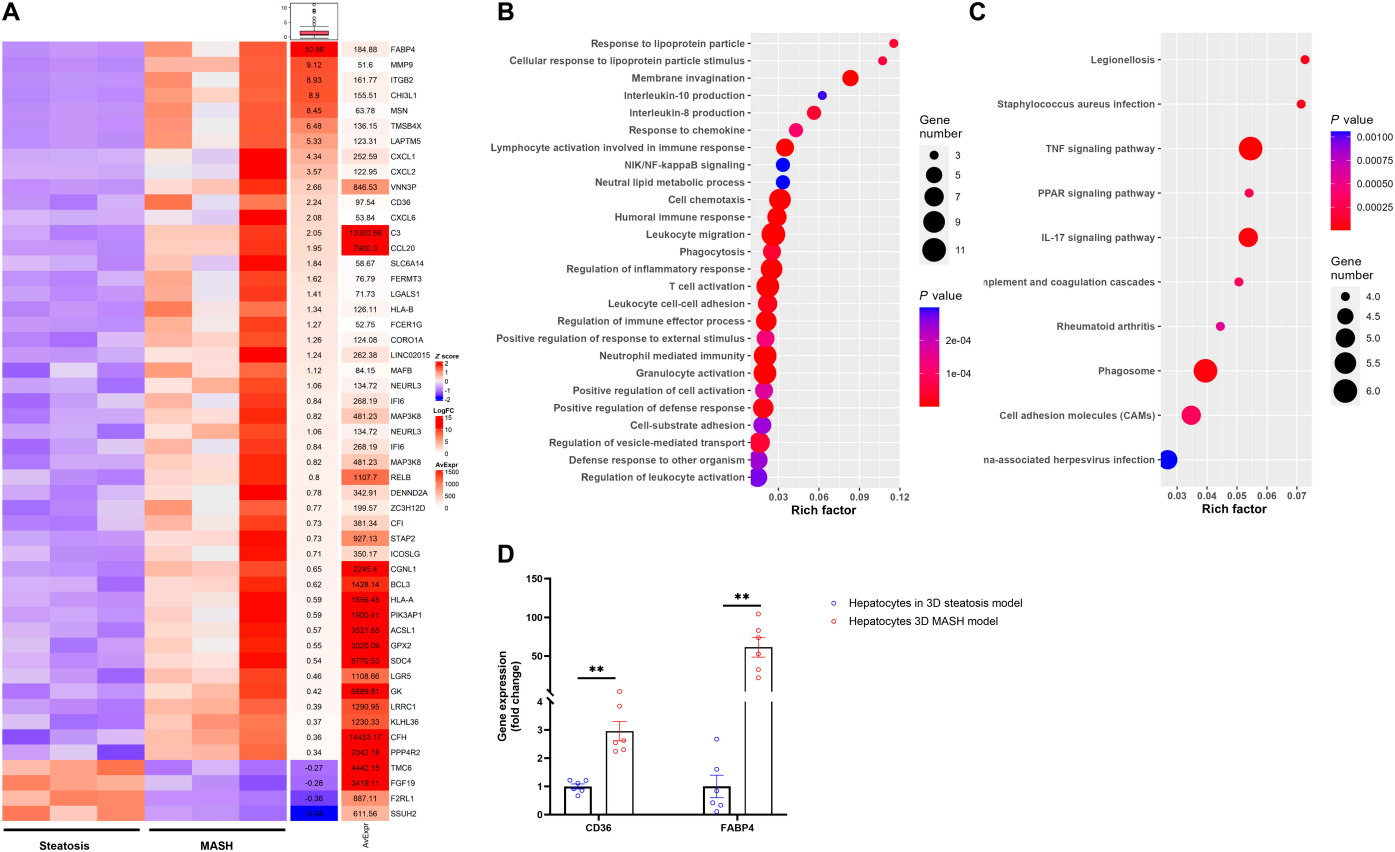
Comparison between 3D MASH and steatosis models revealed the effects of pro-inflammatory macrophages on hepatocytes in MASLD. (A) Heatmap depicting the top 50 DEGs in hepatocytes as determined by log_2_ fold change in the MASH model compared with the steatosis model. (B and C) Gene Ontology (GO) (B) and Kyoto Encyclopedia of Genes and Genomes (KEGG) (C) assessing pathways associated with the DEGs of hepatocytes between 3D MASH and steatosis models. (D) The expression level of critical genes was validated by qPCR. Data are presented by scatterplot with mean ± SEM. **P < 0.01. *N* = 3.

Beyond simulating in vivo MASLD phenotypes and gene expression patterns, the cell carrier-based 3D MASLD models were utilized to explore the mechanistic underpinnings of MASLD progression. A comparative analysis between the 3D steatosis and 3D MASH models shed light on the impact of pro-inflammatory macrophages on hepatocytes in MASLD. A heatmap delineated the top DEGs between these 2 models (Fig. 5A), highlighting critical genes involved in lipid metabolism [*FABP4* (*fatty acid binding protein 4*), *CD36*, and *ACSL1* (*acyl-CoA synthetase long-chain family member 1*)] and inflammation [*CXCL1* (*C-X-C motif chemokine ligand 1*), *CXCL2*, *CXCL6*, *CCL20* (*C-C motif chemokine ligand 20*)*, MAP3K8* (*mitogen-activated protein kinase 8*), *BCL3* (*B cell lymphoma 3*), and *PIK3AP1* (phosphoinositide-3-kinase adaptor protein 1)]. Pathway analyses, incorporating both Gene Ontology (GO) and KEGG databases, indicated significant alterations in lipid metabolism and inflammatory pathways (Fig. 5B and C). The most significant changes in genes in lipid metabolism in RNA-seq were further confirmed by qPCR (Fig. 5D).

## Discussion

In this study, we have developed and validated a series of 3D in vitro MASLD models. These models are based on a cell carrier previously developed in our laboratory [13]. Our MASLD models encompass 3 stages of MASLD: (a) steatosis, characterized by slightly increased lipid metabolism; (b) MASH, marked by macrophage activation and evident cell damage; and (c) fibrosis, where HSC activation is prominent. Our 3D MASLD models, which are established using cell types pertinent to these stages of MASLD, successfully replicate critical phenotypes of the disease, including progressive lipid accumulation and cellular damage. Notably, hepatocytes in our models demonstrate a gene expression profile related to lipid metabolism that is comparable to those in animal MASLD models at corresponding stages. This similarity was confirmed through qPCR and RNA-seq analyses, suggesting that our 3D models may reflect the hepatocellular lipid metabolism seen in MASLD progression. Moreover, separate analyses of macrophages and HSCs within these models verified significant changes associated with MASLD progression, such as inflammation and fibrous tissue formation. Furthermore, our 3D steatosis and MASH models elucidated the influential role of pro-inflammatory macrophages on hepatocyte behavior.

Traditionally, in vitro MASLD models have been established by culturing hepatocyte 2D systems in tissue culture plates and introducing FFAs into the culture medium [22]. This approach, while established and stable for lipid droplet formation in hepatocytes [5], faces challenges due to its inability to mimic the 3D structure, interactions among multiple cell types, and dynamic environment of in vivo conditions. Our MASLD models, utilizing our innovative cell carriers [13], achieve 3D cell culture, interactions among multiple cell types, and dynamic environmental simulation, getting closer to replicating the key characteristics of in vivo systems. As a result, our 3D steatosis model more accurately reflects in vivo conditions, as evidenced by a more representative lipid metabolism gene expression profile and more significant intracellular lipid accumulation compared to traditional 2D models (Table and Fig. 2C and D).

Notably, the absence of significant SREBP1, FASN, and GLUT2 changes in both the 3D steatosis and the mouse steatosis models (Table) indicates cross-model concordance. This pattern likely reflects the early window of FFA exposure, during which hepatocytes predominantly up-regulate oxidative pathways (e.g., PGC1A and CPT1A) rather than strongly suppressing de novo lipogenesis or modulating GLUT2 [23,24]. In addition, differences in cellular composition—hepatocyte-only data from the 3D model versus whole-liver tissue from the mouse model—can dampen direct one-to-one concordance. Taken together, these findings suggest that the lack of significant changes in lipogenic and glucose transporter genes is compatible with stage-dependent regulation, while the overall pathway trends remain aligned across systems.

Due to the significant advantages of 3D culture, various 3D culture methods have emerged in recent years, such as organoids, tissue/organ chips, and cancer spheroids, which serve as crucial platforms for disease modeling, regenerative medicine, and personalized drug screening, highlighting their biomedical significance [25,26]. Recent in vitro MASLD models, based on either microfluidics system/organ-on-a-chip/3D bioprinted models [27–29] or spheroid/organoid/microtissue technologies [12,30,31], often treat and analyze multiple cell types together, which can result in confounding effects among different cell types. For instance, the addition of LPS to stimulate macrophage polarization in coculture systems [11,32] can make it difficult to discern whether observed changes in hepatocytes are due to macrophage interaction or direct LPS effects.

An important feature of our model is the rapid onset of MASLD phenotypes within 24 h, which is significantly faster than most existing 3D models that typically require more than 10 d [33]. This accelerated phenotype formation can be attributed to the relative simplicity of our system compared to other advanced platforms such as microfluidic or organ-on-a-chip models. While these systems provide high biological fidelity, their structural complexity often results in slow nutrient and metabolite diffusion through microchannels or dense 3D matrices, thereby delaying the manifestation of lipid accumulation and inflammatory phenotypes [34,35]. In contrast, our dynamic 3D culture with porous spherical carriers imposes fewer restrictions on FFA exposure, allowing hepatocytes to respond rapidly to metabolic stress, in a manner similar to 2D cultures. However, unlike 2D systems, our coculture design incorporates macrophages and HSCs, enabling physiologically relevant cell–cell interactions while still permitting the separate analysis of individual cell types. This combination of efficient nutrient/FFA exposure, dynamic culture conditions, and cell type-specific analysis contributes to both the reproducibility and the rapid phenotype onset observed in our models.

Apart from creating a 3D dynamic environment for the cells, the other advantage of our MASLD models is that multiple cell types can be treated separately, cocultured together, and analyzed individually. In our models, we could pretreat macrophages with LPS before coculture assembly, ensuring that LPS and other stimulants affect only NPCs, not hepatocytes. This approach allows for a clearer understanding of hepatocyte changes due to cell–cell interactions. At the same time, our novel approach allows us to isolate different types of cells for individual analysis, providing an opportunity to examine the specific role of each cell type in a particular biological process.

Pro-inflammatory macrophages are widely acknowledged as pivotal in the progression of MASLD [20]. Nonetheless, direct evidence elucidating their role in cell–cell interactions has yet to be discovered. This gap was addressed in our research by comparing the steatosis and MASH models, differentiated solely by the presence of pro-inflammatory macrophages. This comparison unveiled the significant impact of these macrophages on hepatocytes. We observed a marked decrease in hepatocyte viability and a concomitant increase in lipid accumulation when cocultured with pro-inflammatory macrophages (Fig. 2), underscoring their contributory role in MASLD progression. RNA-seq further substantiated these findings at the transcriptional level, highlighting alterations in lipid metabolism and inflammatory responses in hepatocytes. Specifically, pathway analysis (Fig. 5B and C) identified significant changes in response to lipoprotein particles, lipid metabolic processes, and the PPAR signaling pathway. Notably, FABP4 and CD36, 2 essential genes in lipid transport [36], were among the most significantly up-regulated genes identified in our heatmap analysis (Fig. 5A) and validated by qPCR (Fig. 5D). This suggests that enhanced lipid transport into hepatocytes, potentially caused by pro-inflammatory macrophage coculture, is a crucial factor in MASLD progression.

CD36 in hepatocytes is primarily responsible for fatty acid uptake and metabolism, and its elevated expression is closely associated with intrahepatic fat accumulation and exacerbation of hepatic inflammation. CD36 is up-regulated in MASLD, promoting hepatocyte uptake of FFAs and exacerbating intrahepatic fat accumulation. CD36 overexpression increases hepatic inflammatory cell infiltration, aggravates hepatic inflammatory responses, and ultimately accelerates the progression of MASLD to MASH [37]. FABP4 plays a crucial role in hepatocytes by regulating fatty acid uptake, transport, and metabolism [38]. Elevated FABP4 expression is typically indicative of hepatic lipid metabolic disorders and the progression of related metabolic diseases [39]. In MASLD, FABP4 overexpression promotes the uptake of FFAs and the synthesis of triglycerides (TGs), leading to hepatic steatosis, while also inhibiting fatty acid β-oxidation, exacerbating lipid accumulation [40]. Additionally, FABP4 activates the nuclear factor κB (NF-κB) signaling pathway, inducing inflammatory responses that contribute to hepatocyte injury and the development of MASH [41]. Inhibiting FABP4 may help alleviate inflammatory responses and improve MASH [42,43]. Clinical studies have shown that increased serum FABP4 levels are closely associated with MASLD, insulin resistance, liver fibrosis, and even HCC [44]. Therefore, elevated FABP4 may serve as a potential biomarker for hepatic lipid metabolic dysfunction and inflammatory status and could be a promising therapeutic target for MASLD and its complications [38]. Results suggest that pro-inflammatory macrophages may induce FABP4 expression in hepatocytes, revealing a novel and significant mechanism in MASLD progression. In terms of inflammation, our pathway analysis (Fig. 5B and C) revealed significant activation in the NF-κB-inducing kinase (NIK)/NF-κB and tumor necrosis factor (TNF) signaling pathways. The up-regulation of specific inflammatory genes (CXCL1, CXCL2, CXCL6, CCL20, MAP3K8, BCL3, and PIK3AP1) further supports the hypothesis that inflammation pathways are stimulated in hepatocytes when cocultured with macrophages. Given the absence of direct cell–cell contact in our coculture system, the macrophage–hepatocyte interaction likely occurs via paracrine effects.

Although our 3D models reproduced most principal lipid metabolism-related gene expression patterns observed in vivo, some differences are expected since our analyses focused on hepatocytes, macrophages, and HSCs, whereas mouse liver tissue contains a broader mixture of cell types (e.g., endothelial cells and cholangiocytes). Moreover, the dynamic expression timing of lipid metabolism-related genes may not fully align between the 3D and in vivo models; for instance, Fig. 3H and I shows broad but not complete concordance in PPAR pathway and fatty acid metabolism genes between the 3D models and the in vivo tissue. These discrepancies likely reflect the simplified cellular environment and shorter culture time in vitro compared with the gradual and multifactorial progression of MASLD in vivo. In addition, although our 3D scaffold culture model offers significant advantages in controllability, reproducibility, and analytical flexibility, it also has certain limitations. Firstly, the cell lines used in our 3D MASLD models, while compatible with our system and effective in demonstrating cell–cell interactions, may only partially replicate in vivo conditions and they are cancer cell lines. Future studies will employ primary cells to enhance the physiological relevance of these models, for example, the primary human hepatocytes [45,46], mouse bone marrow-derived macrophages (BMDMs), human monocyte-derived macrophages (MoDMs), induced pluripotent stem cell (iPSC)-derived macrophages [47], and primary HSCs (or iPSC-derived counterparts) [48,49] to enhance functional maturity [albumin/urea production, inducible cytochrome P450 (CYP) activity], metabolic fidelity of lipid handling and PPAR responses, and the physiological inflammatory/fibrotic signaling of macrophages–HSCs. We anticipate that these upgrades will improve temporal concordance with in vivo MASLD while retaining the model’s strengths in experimental control and cell type-specific readouts. Secondly, the use of mouse liver tissue, which comprises multiple cell types, to validate our in vitro model presents a limitation, given the 3D dynamic model’s focus on single cell types. Advanced techniques like single-cell RNA-seq are anticipated to bridge this gap in future research. Thirdly, compared to organ-on-a-chip systems, our model lacks dynamic fluid flow and mechanical stimulation, which are important for replicating physiological liver conditions such as blood circulation and shear stress. Although our model allows for cell–cell interactions, it does not fully mimic the complex spatial organization and long-term metabolic processes found in organoids or microfluidic platforms. Additionally, while individual cell types can be isolated and analyzed, this approach may not capture the continuous and dynamic interactions between different cell populations over extended periods. Further refinement, such as integrating microfluidic components or optimizing extracellular matrix composition, could enhance the physiological relevance of the model while maintaining its unique advantages in experimental control and analysis.

In summary, our study established a series of novel 3D dynamic coculture models for various MASLD stages, including steatosis, MASH, and fibrosis. These models successfully mimic both phenotypic and transcriptional changes observed in well-established mouse MASLD models.

## Data Availability

The RNA-seq data generated in this study have been deposited in National Center for Biotechnology Information (NCBI) under the accession number (BioProject ID: PRJNA1264068).
