# Supplementary material for: Three-Dimensional Dynamic Cell Models for Metabolic Dysfunction-Associated Steatotic Liver Disease Progression
Source: BME Front. 2025 Sep 30;6:0181. doi: 10.34133/bmef.0181 (PMC12480745; doi:10.34133/bmef.0181)
Supplement: Supplementary 1 — Figs. S1 to S6 Tables S1 and S2 [file bmef.0181.f1.pdf]

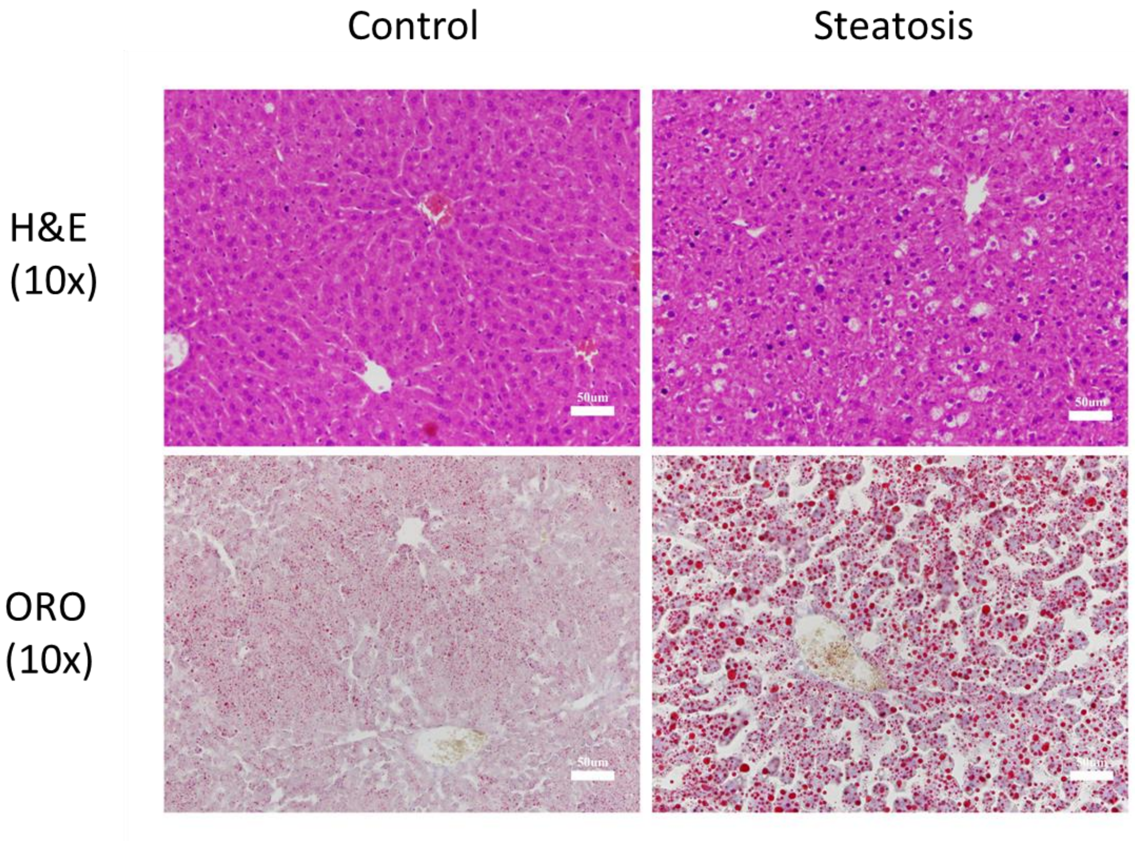

Figure S1. Representative liver histology of the Steatosis animal model used in this study. H&E, Hematoxylin and eosin staining; ORO, oil-red-o staining.

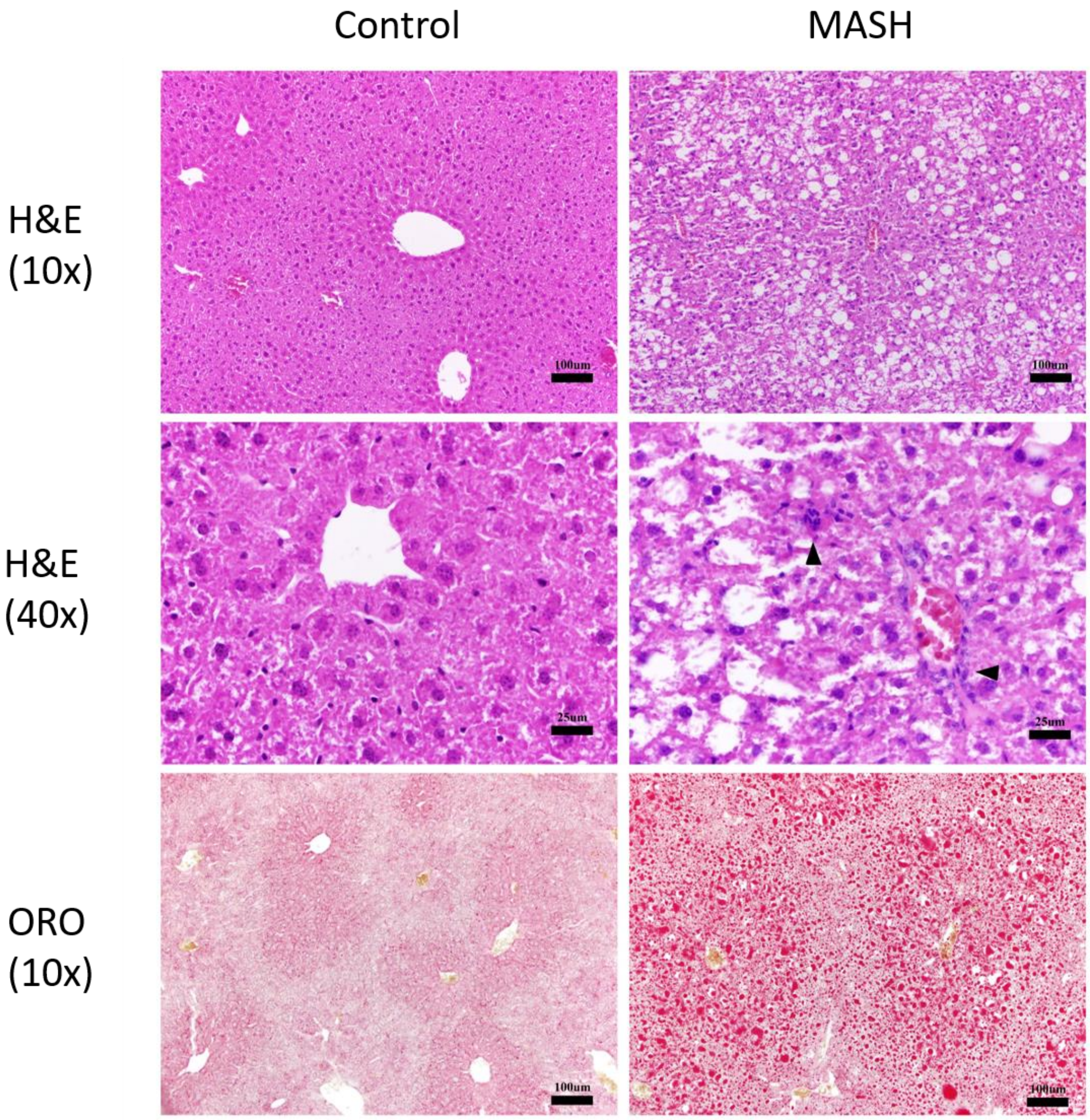

Figure S2. Representative liver histology of the NASH animal model used in this study.

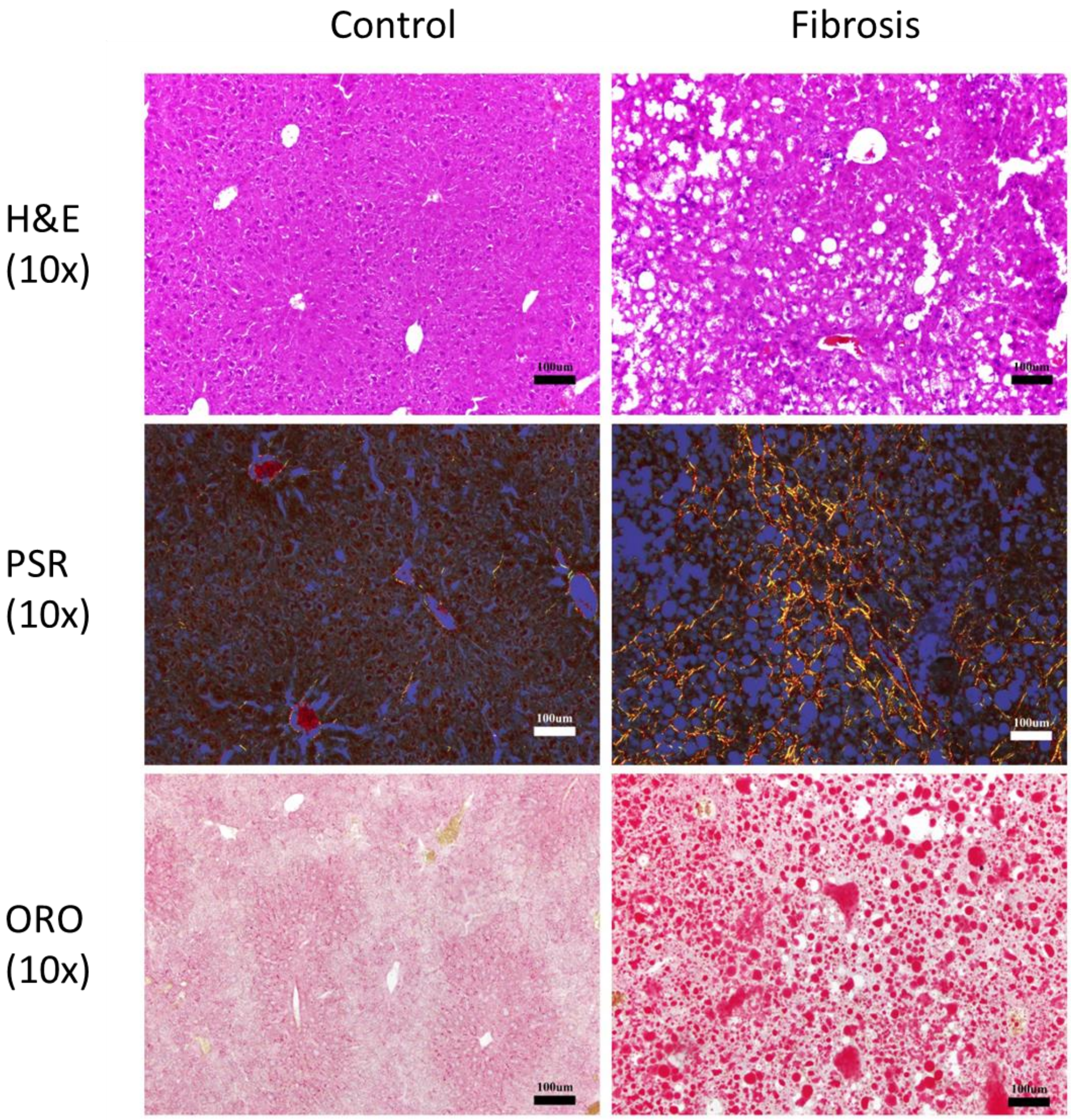

Figure S3. Representative liver histology of the Fibrosis animal model used in this study. PSR, picrosirius red staining.

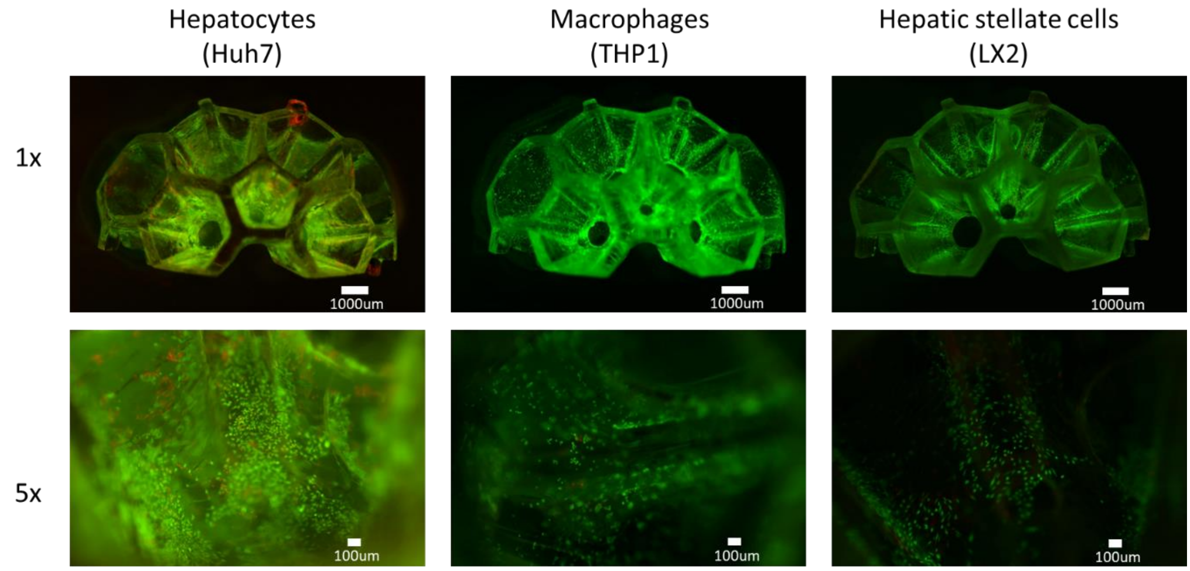

Figure S4. FDA/PI staining on the cell carriers with cells following seeding.

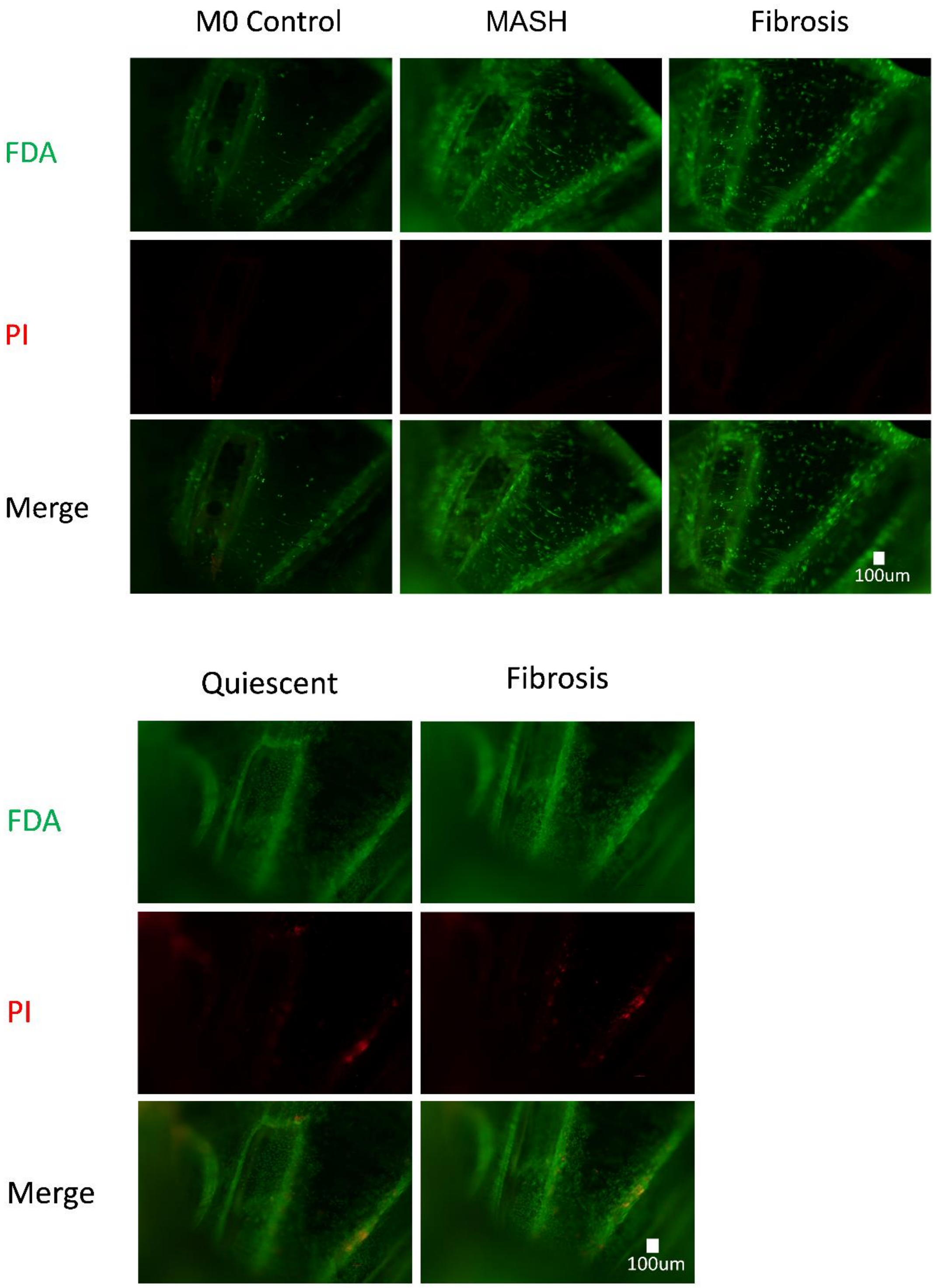

Figure S5. FDA/PI staining on macrophages (top) and hepatic stellate cells (bottom) in different conditions.

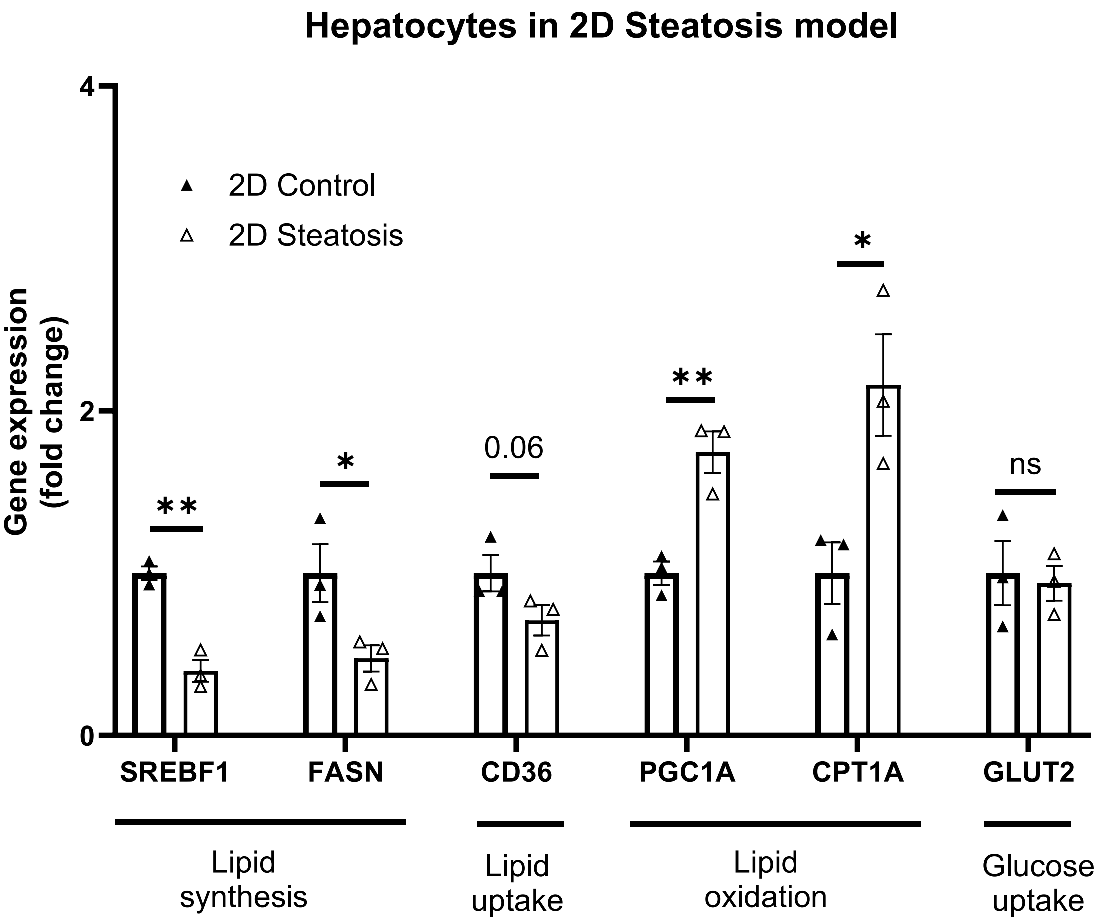

Figure S6. The gene expression level change in the 2D Steatosis model compared to its control. Gene expression fold change was measured by qPCR. Data were presented by scatter plot with mean, n = 3. *P < 0.05; **P < 0.01; ns, non-significant.

Table S1. qPCR primers sequences (mouse)

	Forward 5'--3'	Reverse 5'--3'
Actb	CTGAATGGCCCAGGTCTGA	CCCTGGCTGCCTCAACAC
Srebf1	TGACCCGGCTATTCCGTGA	CTGGGCTGAGCAATACAGTTC
Fasn	GGAGGTGGTGATAGCCGGTAT	TGGGTAATCCATAGAGCCCAG
Cd36	ATGGGCTGTGATCGGAACTG	GTCTTCTCAATAAGCATGTCTCC
Cpt1a	CTATGCGCTACTCGCTGAAGG	GGCTTTCGACCCGAGAAGA
Pgc1a	AGCCGTGACCACTGACAACGAG	GCTGCATGGTTCTGAGTGCTAAG
Glut2	TCAGAAGACAAGATCACCGGA	GCTGGTGTGACTGTAAGTGGG
Col1a1	GCTCCTCTTAGGGGCCACT	CCACGTCTCACCATTGGGG
Col3a1	CTGTAACATGGAAACTGGGGAAA	CCATAGCTGAACTGAAAACCACC
Tnf	CTGAACTTCGGGGTGATCGG	GGCTTGTCACTCGAATTTTGAGA
Il1b	TGGAGAGTGTGGATCCCAAG	GGTGCTGATGTACCAGTTGG
Cd80	AAAAGAAGGAAAGAGGAACGTATGAA	CCGGAAGCAAAGCAGGTAATC
Cd86	CTGCTCATCATTGTATGTCAC	ACTGCCTTCACTCTGCATTTG
Ccl2	TTAAAAACCTGGATCGGAACCAA	GCATTAGCTTCAGATTTACGGGT
Tgfb	CAGTACAGCAAGGTCCTTGC	ACGTAGTAGACGATGGGCAG
Cd206	CTCTGTTCAGCTATTGGACGC	CGGAATTTCTGGGATTCAGCTTC
Cd163	GGTGGACACAGAATGGTTCTTC	CCAGGAGCGTTAGTGACAGC

Table S2. qPCR primers sequences (human)

	Forward 5'--3'	Reverse 5'--3'
ACTB	TCCAGCTCCTCCCTGGAG	ACAGGACTCCATGCCCAG
SREBF1	CGGAACCATCTTGGCAACAGT	CGCTTCTCAATGGCGTTGT
FASN	AAGGACCTGTCTAGGTTTGATGC	TGGCTTCATAGGTGACTTCCA
CD36	CTTTGGCTTAATGAGACTGGGAC	GCAACAAACATCACCACACCA
PGC1A	TCTGAGTCTGTATGGAGTGACAT	CCAAGTCGTTCACATCTAGTTCA
CPT1A	TCCAGTTGGCTTATCGTGGTG	TCCAGAGTCCGATTGATTTTTGC
GLUT2	GCTGCTCAACTAATCACCATGC	TGGTCCCAATTTTGAAAACCCC
COL1A1	GAGGGCCAAGACGAAGACATC	CAGATCACGTCATCGCACAAC
COL3A1	GCCAAATATGTGTCTGTGACTCA	GGGCGAGTAGGAGCAGTTG
TNFA	GAGGCCAAGCCCTGGTATG	CGGGCCGATTGATCTCAGC
IL1B	ATGATGGCTTATTACAGTGGCAA	GTCGGAGATTCGTAGCTGGA
CD80	AAACTCGCATCTACTGGCAAA	GGTTCTTGTACTCGGGCCATA
CD86	CTGCTCATCTATACACGGTTACC	GGAAACGTCGTACAGTTCTGTG
CCL2	CAGCCAGATGCAATCAATGCC	TGGAATCCTGAACCCACTTCT
TGFB1	GGCCAGATCCTGTCCAAGC	GTGGGTTTCCACCATTAGCAC
CD206	GGGTTGCTATCACTCTCTATGC	TTTCTTGTCTGTTGCCGTAGTT
CD163	TTTGTCAACTTGAGTCCCTTCAC	TCCCGCTACACTTGTTTTCAC
